# (+)-Catechin protects dermal fibroblasts against oxidative stress-induced apoptosis

**DOI:** 10.1186/1472-6882-14-133

**Published:** 2014-04-08

**Authors:** Tomoko Tanigawa, Shigeyuki Kanazawa, Ryoko Ichibori, Takashi Fujiwara, Takuya Magome, Kenta Shingaki, Shingo Miyata, Yuki Hata, Koichi Tomita, Ken Matsuda, Tateki Kubo, Masaya Tohyama, Kenji Yano, Ko Hosokawa

**Affiliations:** 1 Department of Plastic Surgery, Osaka University Graduate School of Medicine, Suita-shi, Osaka, Japan; 2 Department of Child Development and Molecular Brain Science, United Graduate School of Child Development, Osaka University, Suita-shi, Osaka, Japan; 3 Department of Research & Development Noevir Co., Ltd. Higashiomi, Shiga, Japan; 4 Division of Molecular Brain Science, Research Institute of Traditional Asian Medicine, Kinki University, Osakasayama, Osaka, Japan; 5 Division of Plastic and Reconstructive Surgery, Niigata University Graduate School of Medicine, Niigata-shi, Niigata, Japan

**Keywords:** Catechin, Fibroblast, Apoptosis, Oxidative stress

## Abstract

**Background:**

Oxidative stress has been suggested as a mechanism underlying skin aging, as it triggers apoptosis in various cell types, including fibroblasts, which play important roles in the preservation of healthy, youthful skin. Catechins, which are antioxidants contained in green tea, exert various actions such as anti-inflammatory, anti-bacterial, and anti-cancer actions. In this study, we investigated the effect of (+)-catechin on apoptosis induced by oxidative stress in fibroblasts.

**Methods:**

Fibroblasts (NIH3T3) under oxidative stress induced by hydrogen peroxide (0.1 mM) were treated with either vehicle or (+)-catechin (0–100 μM). The effect of (+)-catechin on cell viability, apoptosis, phosphorylation of c-Jun terminal kinases (JNK) and p38, and activation of caspase-3 in fibroblasts under oxidative stress were evaluated.

**Results:**

Hydrogen peroxide induced apoptotic cell death in fibroblasts, accompanied by induction of phosphorylation of JNK and p38 and activation of caspase-3. Pretreatment of the fibroblasts with (+)-catechin inhibited hydrogen peroxide-induced apoptosis and reduced phosphorylation of JNK and p38 and activation of caspase-3.

**Conclusion:**

(+)-Catechin protects against oxidative stress-induced cell death in fibroblasts, possibly by inhibiting phosphorylation of p38 and JNK. These results suggest that (+)-catechin has potential as a therapeutic agent for the prevention of skin aging.

## Background

Skin wrinkles and sagging are important factors defining skin youthfulness. Development of methods to reduce skin wrinkles and prevent sagging skin has become an important research topic in aesthetic and anti-aging medicine. Skin wrinkles and sagging are reported to be influenced by the amount of collagen, elastin, and hyaluronic acid [1]. Fibroblasts play a key role in the production of these extracellular matrix components in the skin. Skin aging is the consequence of reduced numbers of fibroblasts, lower levels of extracellular matrix proteins, and decreased skin elasticity and tonus, thereby resulting in the formation of wrinkles [2]. Therefore, maintaining the population of dermal fibroblasts is important for both preventing and treating age-related skin changes.

Oxidative stress has been indicated in a variety of pathological processes, such as atherosclerosis, diabetes, neurodegenerative diseases, and aging. Reactive oxygen species induce DNA damage, intracellular lipid peroxidation, and abnormal protein oxidation reactions, all of which result in cell damage. Oxidative stress also promotes skin aging [3]; it reduces the number of skin fibroblasts by inducing apoptosis and decreasing their regenerative capacity, which in turn leads to increased skin sagging. Therefore, suppression of oxidative stress-induced apoptosis in skin fibroblasts is a potential treatment and prevention strategy for maintaining healthy youthful skin.

**Figure 1:**
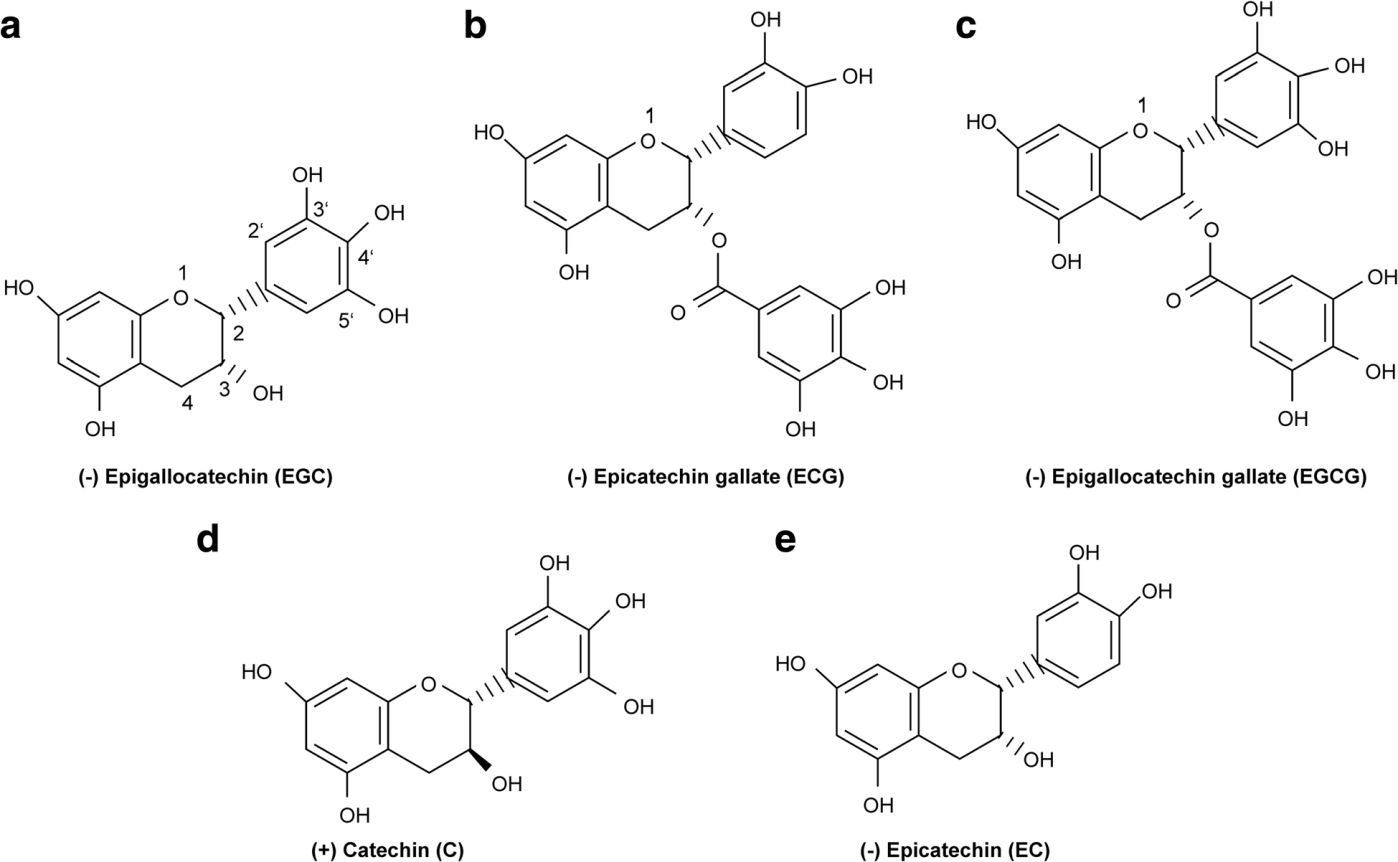
**Chemical Structures of catechins.** Structures of (−)-epigallocatechin (EGC) **(a)**, (−)-epicatechin gallate (ECG) **(b)**, (−)-epigallocatechin gallate (EGCG) **(c)**, (+)-catechin(C) **(d)**, and (−)-epicatechin (EC) **(e)**.

Green tea, which is routinely consumed in Japan and China, is widely known as a healthy drink containing various antioxidants, vitamins, and minerals. Catechins, including (−)-epigallocatechin gallate (EGCG), (−)-epigallocatechin (EGC), (−)-epicatechin gallate (ECG), and (−)-epicatechin (EC) (Figure 1), account for approximately 10% of the dry weight of green tea leaves. Catechins are thought to not only possess antioxidant effects to control active oxygen [4-7] but also exert various actions, such as anti-inflammatory [8], antibacterial [9,10], and anti-cancer [11-13] actions.

In this study, we demonstrate that (+)-catechin has an inhibitory effect against oxidative stress-induced apoptosis in fibroblasts, accompanied by suppression of phosphorylation of p38 and c-Jun terminal kinases (JNK), both of which play an important role in intracellular apoptotic signaling induced by oxidative stress.

## Methods

### Cell culture

NIH 3T3 fibroblasts were used for all experiments. Cells were cultured in Dulbecco’s Modified Eagle Medium (DMEM; Life Technologies CA, USA) containing 10% fetal bovine serum (FBS), 100 U/ml penicillin, and 100 μg/ml streptomycin (Life Technologies) in a humidified incubator at 37°C with 5% CO_2_. All experiments were performed in triplicate.

### Cell viability assay

Cell survival was determined using the 3-(4,5-dimethylthiazole-2-yl)-2,5-diphenyltetrazolium bromide (MTT) assay (CellTiter 96^®^ AQueous One Solution Cell Proliferation Assay; Promega, WI, USA). Fibroblasts were plated at a density of 5,000 cells per well on 96-well plates and incubated for 24 h in 100 μl of DMEM containing 10% FBS. After incubation with serum-free medium for 24 h, cells were treated for 30 min with various concentrations of (+)-catechin (0–400 μM; Sigma Aldrich, PA, USA), and then subjected to oxidative stress induction with 0.1 mM hydrogen peroxide (H_2_O_2_). After 24 h, 20 μl of One Solution Reagent was added into each well and incubated at 37°C for 2 h in a humidified, 5% CO_2_ atmosphere. The production of formazan by viable cells was detected by measuring the absorbance at 490 nm using a 96-well plate reader.

Another series of experiments were conducted to compare cytotoxicity between (+)-catechin and EGCG. Fibroblasts were treated with various concentrations of (+)-catechin or EGCG (0–400 μM; Sigma Aldrich) without H_2_O_2_ for 24 h and the cells were then subjected to MTT assay.

### TUNEL staining

Apoptosis was determined by terminal deoxynucleotidyl transferase (TdT)-mediated dUTP-biotin nick end labeling (TUNEL) using the *In Situ* Cell Death Detection Kit TMR Red (Roche, Mannheim, Germany), according to the manufacturer’s instructions. In brief, fibroblasts were maintained in DMEM containing 10% FBS for 2 days and then cultured in serum-free DMEM. Oxidative stress was induced by addition of 0.1 mM H_2_O_2_ prior to treatment with 10 μM (+)-catechin or vehicle. After 24 h of incubation with H_2_O_2_ and (+)-catechin or vehicle, cells were fixed with 4% paraformaldehyde in phosphate-buffered saline (PBS) (pH 7.4) for 60 min at room temperature, followed by five washes with PBS. Next, permeabilization was performed by incubation with 0.1% Triton X-100 in PBS for 10 min, and cells were mixed with TUNEL reaction mixtures containing TdT and tetramethylrhodamine (TMR) red-labeled nucleotides for 1 h. Coverslips were mounted onto slides using VECTASHIELD Mounting Medium with 4′,6-diamidino-2-phenylindole dihydrochloride (Vector Laboratories, Peterborough, England). Fluorescence images were taken using a microscope (IX-70; Olympus) equipped with a charge-coupled device camera (CoolSNAP HQ; Nippon Roper, Chiba, Japan). For each experiment, 100 cells were randomly selected, and the percentage of TUNEL-positive cells was measured.

### Western blot analysis

Cultured fibroblasts were serum-starved for 24 h in serum-free DMEM and then incubated with 10 μM (+)-catechin for 30 min prior to oxidative stress induction by 0.1 mM H_2_O_2_. After H_2_O_2_ challenge for 1 h, cells were harvested and lysed in radioimmunoprecipitation assay buffer containing 1 mM Na_3_VO_4_, 1 mM NaF, and Protease Inhibitor Cocktail (Roche Diagnostics, Basel, Switzerland) for 20 min at 4°C. After centrifugation at 15,000 × *g* for 15 min at 4°C, proteins were separated by sodium dodecyl sulfate-polyacrylamide gel electrophoresis and transferred onto Immobilon-P Transfer Membranes (Millipore Japan, Tokyo, Japan). Membranes were incubated for 60 min in Tris-buffered saline containing 5% skim milk and 0.05% Tween-20 and then blotted with the following primary antibodies at 4°C overnight: anti-phospho-JNK (1:1,000), anti-JNK (1:1,000), anti-phospho-p38 (1:1,000), anti-p38 (1:1,000), anti-cleaved caspase-3 (1:200), and anti-caspase-3 antibodies (1:200). All antibodies were purchased from Cell Signaling Technology, MA, USA. Next, membranes were incubated for 1 h with an anti-mouse or anti-rabbit HRP-linked secondary antibody (1:2,000; Cell Signaling Technology). Reaction products were visualized by chemiluminescence detection using the ECL Western Blotting Detection System (GE Healthcare, Piscataway, NJ, USA). Quantification of relative band densities was performed by densitometry using Image J software (National Institutes of Health, Bethesda, MD, USA).

### Statistical analysis

All data shown are expressed as the mean ± SE of three independent experiments. Data from each experiment were normalized to the respective control sample. Differences between conditions were analyzed by Student’s *t* test. Multiple-group comparisons were performed using a one-way analysis of variance, followed by Tukey’s post hoc test. P < 0.05 was considered statistically significant.

## Results

### Catechin increases the viability of fibroblasts

**Figure 2:**
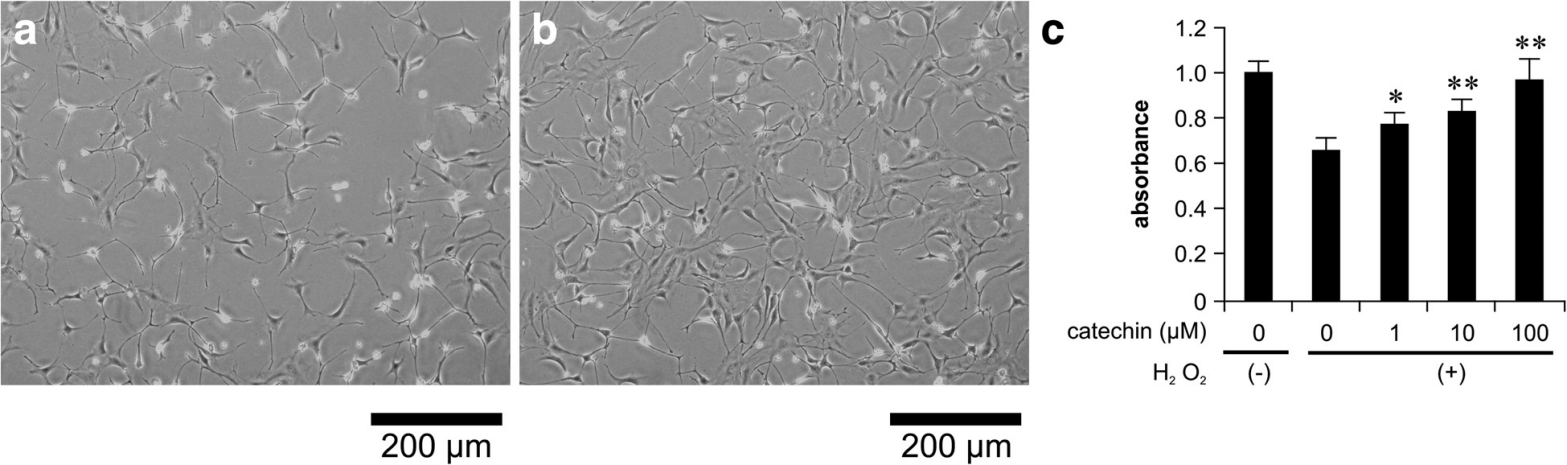
**Inhibitory effect of (+)-catechin on oxidative stress-induced cell death in fibroblasts.** After incubation with serum-free medium for 24 h, fibroblasts were treated for 30 min with (+)-catechin (0–400 μM) and then subjected to oxidative stress induction with 0.1 mM hydrogen peroxide (H_2_O_2_). After 24 h, cell viability was evaluated. **(a)** Representative microscopic images of cell death induced by oxidative stress **(a)** and inhibitory effect of (+)-catechin on oxidative stress-induced cell death **(b)**. Cell viability was assessed by the MTT assay **(c)**. Data are expressed as the mean ± SEM. *P < 0.05, **P < 0.01.

Oxidative stress is known to promote fibroblast cell death [14]. To analyze the effect of (+)-catechin on the viability of fibroblasts in response to oxidative stress, cells cultured with various concentrations (0–100 μM) of catechin were subjected to oxidative stress induction by 0.1 mM H_2_O_2_. The cell numbers were analyzed after 24 h. Microscopic observation and MTT assay showed that H_2_O_2_ induced-oxidative stress reduced cell viability, whereas (+)-catechin suppressed the effect of H_2_O_2_-induced oxidative stress on cell viability in a concentration-dependent manner (Figure 2a-c).

**Figure 3:**
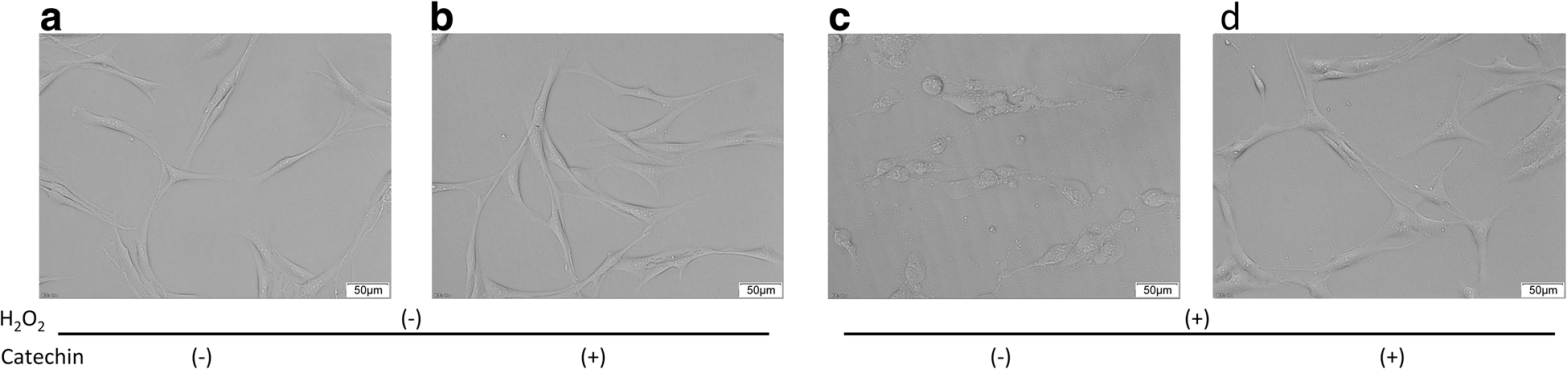
**Microscopic images of H**_**2**_**O**_**2**_**-induced apoptotic cell death and inhibitory effect of (+)-catechin against H**_**2**_**O**_**2**_**-induced cell death. (a)** The image of non-loading control cells. After incubation with serum-free medium for 24 h, fibroblasts were treated for 30 min with 10 μM (+)-catechin **(b)**, **(d)**. And then subjected to oxidative stress induction with 0.1 mM H_2_O_2_**(c)**, **(d)**. After 24 h, microscopic morphological changes were evaluated.

As shown in Figure 3, microscopic evaluation of the morphological changes showed that H_2_O_2_ supplementation in the culture media induced apoptotic cell death characterized by shrinkage of the cell body, whereas treatment with (+)-catechin attenuated H_2_O_2_-induced cell death.

### (+)-Catechin inhibits oxidative stress-induced apoptosis in fibroblasts

**Figure 4:**
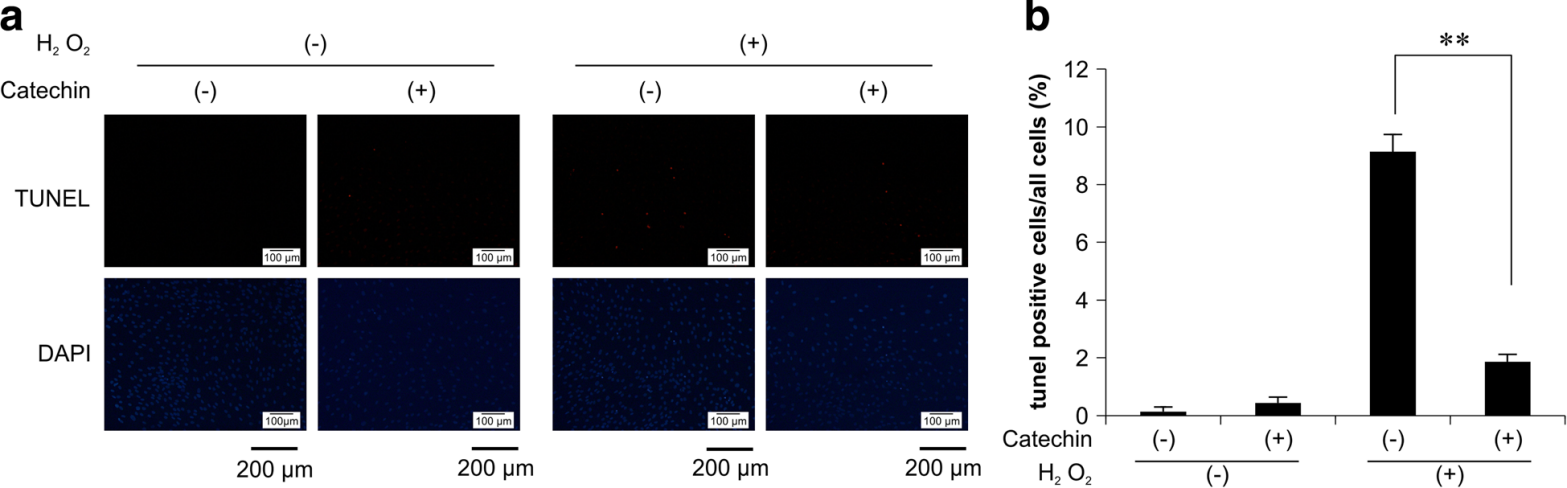
**Inhibitory effect of (+)-catechin on oxidative stress-induced apoptosis in fibroblasts.** After incubation with serum-free medium for 24 h, fibroblasts were treated for 30 min with 10 μM (+)-catechin and then subjected to oxidative stress induction with 0.1 mM hydrogen peroxide (H_2_O_2_). After 24 h, apoptosis was evaluated by TUNEL staining. **(a)** Microscopic findings of TUNEL staining for detection of apoptotic cells. **(b)** For evaluation of apoptosis, 100 cells were randomly selected and the percentage of TUNEL-positive cells was measured. Data are expressed as the mean ± SEM. **P < 0.01.

To determine whether (+)-catechin has an inhibitory effect on oxidative stress-induced apoptosis in fibroblasts, we assessed the apoptosis of fibroblasts in either the presence or absence of (+)-catechin by TUNEL staining. (+)-Catechin (10 μM)-treated fibroblasts showed significant decreases in the percentage of cells positive for TUNEL staining, compared to vehicle-treated cells (9.14% ± 0.6% vs. 1.86% ± 0.3%; Figure 4).

### Effect of catechin on the activation of caspase-3 by H_2_O_2_-induced oxidative stress in fibroblasts

**Figure 5:**
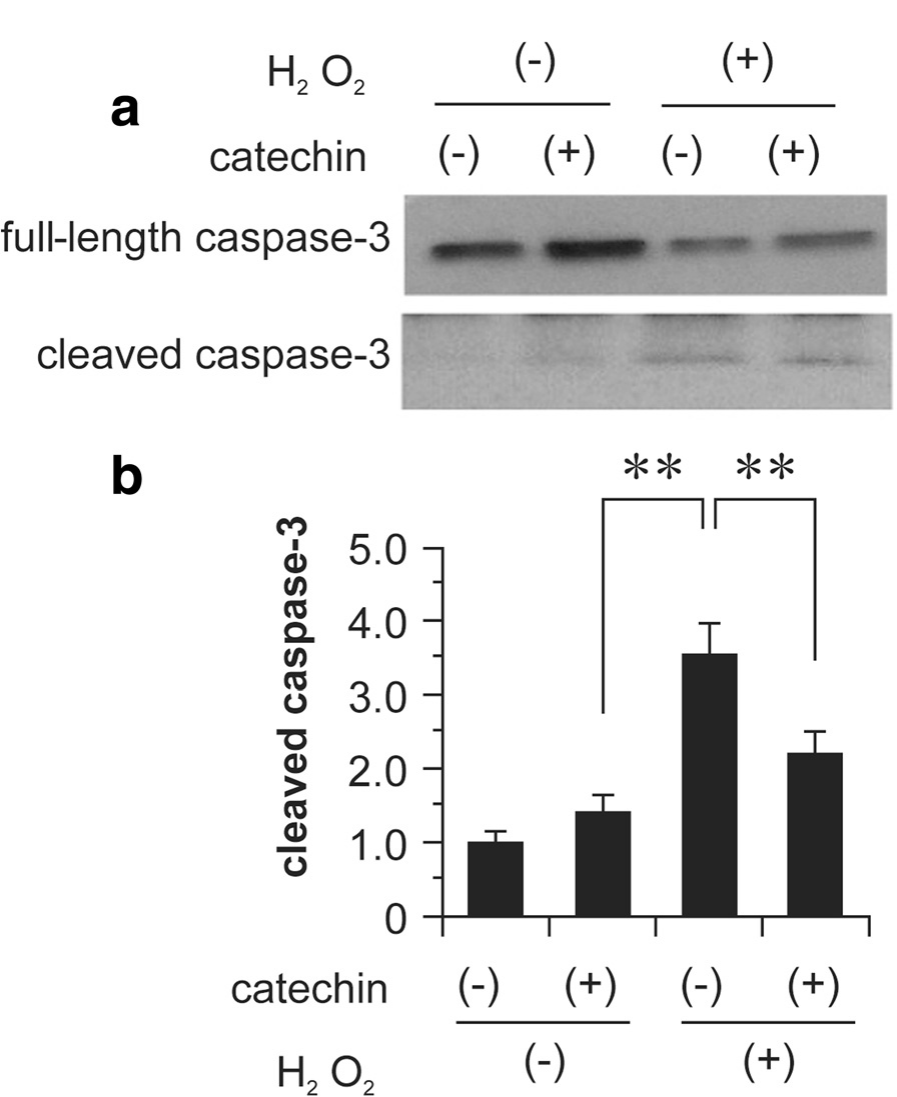
**Effect of (+)-catechin on activation of caspase-3 by H**_**2**_**O**_**2**_**-induced oxidative stress in fibroblasts.** After incubation with serum-free medium for 24 h, fibroblasts were treated for 30 min with 10 μM (+)-catechin and then subjected to oxidative stress induction with 0.1 mM hydrogen peroxide (H_2_O_2_). After 1 h, activation of caspase-3 was determined by SDS-PAGE and western blotting analysis using an anti-cleaved caspase-3 antibody. **(a)** Representative images of western blot analysis for cleaved and total caspase-3. **(b)** Expression levels of cleaved caspase-3 were normalized to those of total caspase-3. Data are expressed as the mean ± SEM. **P < 0.01.

Western blotting analysis using an anti-cleaved caspase-3 antibody showed that the level of cleaved caspase-3 induced by H_2_O_2_ was reduced by treatment with 10 μM (+)-catechin (Figure 5). These results suggest that (+)-catechin inhibits caspase-3-dependent apoptosis induced by oxidative stress in fibroblasts.

### (+)-Catechin inhibits phosphorylation of p38 and JNK induced by oxidative stress

**Figure 6:**
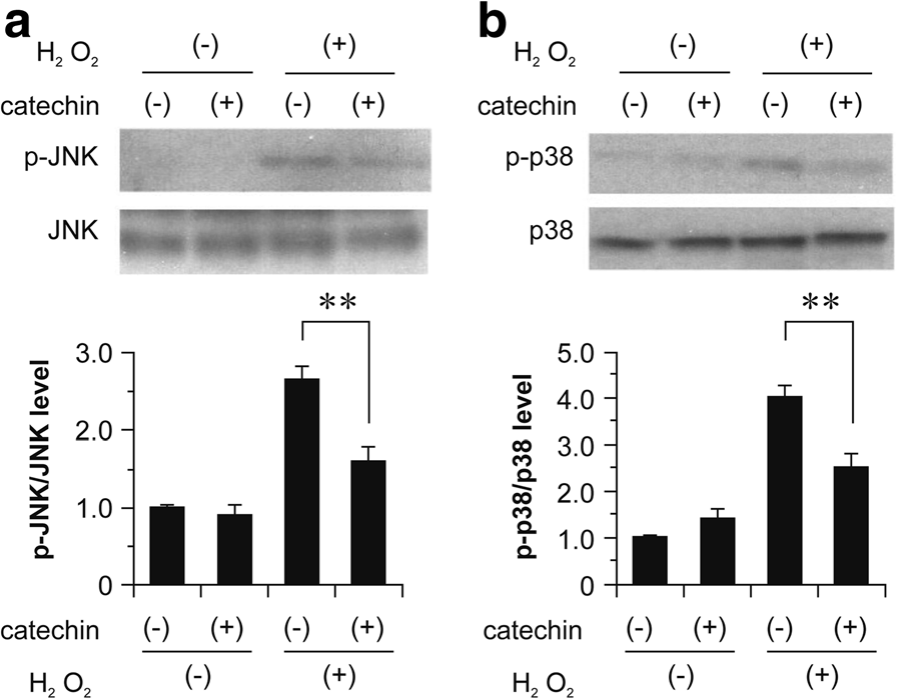
**Inhibitory effect of (+)-catechin on phosphorylation of p38 and JNK induced by oxidative stress in fibroblasts.** After incubation with serum-free medium for 24 h, fibroblasts were treated for 30 min with 10 μM (+)-catechin and then subjected to oxidative stress induction with 0.1 mM hydrogen peroxide (H_2_O_2_). After 1 h, cells were collected, and phosphorylation of p38 and JNK was determined by SDS-PAGE and western blotting analysis using anti-phospho p38 and anti-phospho JNK antibodies. **(a)** Results of western blotting for phosphorylation of **(a)** p38 and **(b)** JNK. Phosphorylation levels of p38 and JNK were normalized to those of total p38 and JNK, respectively. Data are expressed as the mean ± SEM. **P < 0.01.

To further investigate the underlying mechanism by which (+)-catechin inhibits oxidative stress-induced apoptosis in fibroblasts, we determined whether oxidative stress-induced phosphorylation of JNK and p38 was inhibited by treatment with 10 μM (+)-catechin. The results clearly show that H_2_O_2_-induced phosphorylation of p38 and JNK was suppressed by (+)-catechin treatment (Figure 6).

### (+)-catechin is less cytotoxic than EGCG in fibroblasts

**Figure 7:**
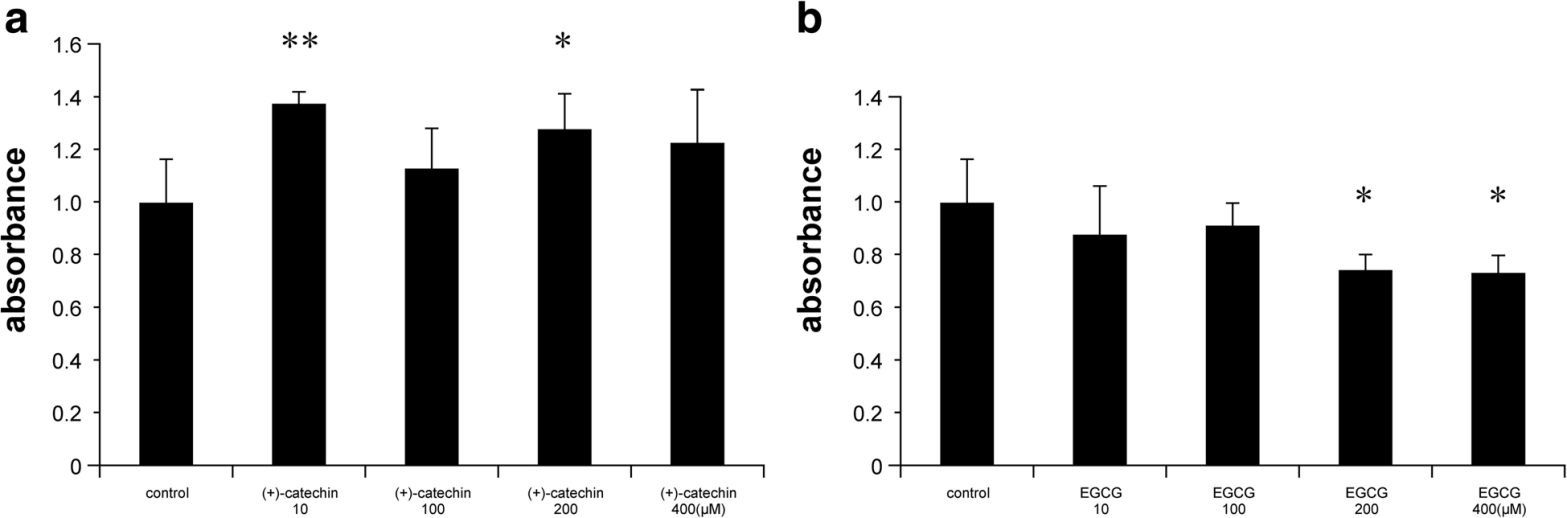
**Comparative evaluation of cytotoxicity between (+)-catechin and EGCG in fibroblasts.** Fibroblasts were treated with PBS (control) or the indicated concentrations of **(a)** (+)-catechin and **(b)** EGCG for 24 h. Cell viability was assessed by the MTT assay. Data are expressed as the mean ± SEM. *P < 0.05 and **P < 0.01.

The MTT assay showed that fibroblasts were viable when incubated with high concentrations of (+)-catechin. In contrast, EGCG at 200 and 400 μM significantly decreased cell viability (Figure 7).

## Discussion

In the present study, we demonstrate an inhibitory effect of (+)-catechin on oxidative stress-induced apoptosis in fibroblasts, accompanied by amelioration of the phosphorylation of p38 and JNK induced by oxidative stress.

We focused on fibroblasts because they participate in skin maintenance and renewal. In the skin, fibroblasts play a key role in the production of extracellular matrix components, including collagen, elastin, and hyaluronic acid. In clinical aesthetic medicine, epidermal or intradermal injection of hyaluronic acid is performed to obtain glossy and healthy skin (microinjections of hyaluronic acid, vitamins, minerals, and amino acids into the superficial layer of the skin) [15]. Other techniques, such as implanting activated fibroblasts in the skin, are also known to revive the skin to be glossy and healthy (intradermal injection of cultivated skin fibroblasts into wrinkles) [16-18]. However, these therapies are associated with a high cost and may provoke adverse events, including misplacement, allergy, nodules, necrosis, abscesses, and rejection. In contrast, the use of health supplements, such as green tea and food-derived active substances, is a safer and beneficial anti-aging method.

The integrity and functions of the skin barrier may be impaired by excessive exposure to allergens, chemicals, ultraviolet light, and dehydration. Failure of the skin barrier would subsequently lead to infections with pathogens and result in inflammatory responses. Locally produced reactive oxygen species are also known to inhibit the growth of epithelial cells and fibroblasts by inducing apoptosis and inhibiting collagen and hyaluronic acid production, all of which have been implicated in aging processes leading to skin wrinkles and sagging. Our present study suggests that (+)-catechin is a potential candidate for suppressing oxidative stress-induced apoptosis of skin fibroblasts, which may in turn reverse the reduction of fibroblast-derived production of collagen and hyaluronic acid. Other reports suggest that EGCG, another type of catechin, is also a potential candidate for suppressing oxidative stress-induced apoptosis of skin fibroblasts [19]; however, our present study showed that (+)-catechin is less cytotoxic than EGCG, suggesting that for therapeutic and preventive purposes (+)-catechin may be superior to EGCG.

To elucidate the underlying mechanisms by which (+)-catechin inhibits oxidative stress-induced apoptosis in fibroblasts, we focused on the effects of (+)-catechin on the phosphorylation of p38 and JNK, both of which are key molecules for oxidative stress-induced apoptosis [14]. JNK and p38 belong to the family of stress kinases and have been shown to be required for biological stress responses, such as apoptosis induced by UV, radiation, oxidative stress, heat shock, and tumor necrosis factor (TNF)-α stimulation. It has been reported that H_2_O_2_ signaling through TNF receptor 1 selectively activates JNK and p38 [20,21]. JNK plays an important role in controlling cell death and is known to affect the function of Bcl-2 family molecules, which suppress apoptosis. Specifically, phosphorylation of Bcl-2 by JNK results in the inhibition of Bcl-2 function and therefore induces the activation of apoptosis [20,21]. In contrast, p38 MAPK is known to be involved in the activation of apoptosis-modulating proteins, such as Fas and Bax [21]. Collectively, our present study suggests that (+)-catechin exerts anti-apoptotic effects against oxidative stress by inhibiting the phosphorylation of p38 and JNK. The precise mechanisms by which (+)-catechin suppresses the phosphorylation of JNK and p38 will be a future research topic.

Although (+)-catechin was found to exert anti-apoptotic effects in the present study, previous reports have shown both pro-apoptotic and anti-apoptotic effects of catechins. In particular, EGCG, a molecule in the same catechin group, was suggested to play a role in growth inhibition and apoptosis induction in a variety of cancer cells [22]. In contrast, EGCG was reported to have an anti-apoptotic effect in renal mesangial cells [23] and endothelial cells [24], similar to our results in the present study. Therefore, we speculate that the effect of catechins on apoptosis may vary according to cell type and the nature of pathogenesis. Given the different cell-specific responses of catechins, it is important to establish an appropriate strategy for using catechins for treatment and prevention of various diseases. It would be ideal for catechins have suppressive actions against cancers and protective effects for organs such as the kidneys and cardiovascular system. Accumulating evidence on the preventive effect of catechins and green tea against various systemic diseases, including cancers, diabetes, and hypertension, suggests little potential harm to human health from high consumption of catechins and green tea for maintenance of skin beauty.

## Conclusions

(+)-Catechin exerts preventive effects against oxidative stress-induced apoptosis in fibroblasts. The underlying mechanism may involve the inhibition of p38 and JNK phosphorylation. As a safe green tea-derived antioxidant, (+)-catechin could be suitable for long-term prevention of oxidative stress-induced skin aging, considering the action of skin fibroblasts on the preservation of healthy, youthful skin.

## Abbreviations

EGCG: (−)-epigallocatechin gallate; EGC: (−)-epigallocatechin; ECG: (−)-epicatechin gallate; EC: (−)-epicatechin; JNK: c-Jun terminal kinase MTT, 3-(4,5-dimethylthiazole-2-yl)-2,5-diphenyltetrazolium bromide; TdT: Terminal deoxynucleotidyl transferase; TMR: Tetramethylrhodamine; TUNEL: Terminal deoxynucleotidyl transferase (TdT)-mediated dUTP-biotin nick end labeling.

## Competing interests

The authors declare that they have no competing interests.

## Authors’ contributions

TT designed and performed the study and wrote the manuscript. SK designed and performed the study and revised the manuscript. RY, TF, TK, KY, and KH helped to perform the study. TM, YH, KT, KM, KS, SM, and MT provided technical support. All authors read and approved the final manuscript.

## Pre-publication history

The pre-publication history for this paper can be accessed here:

http://www.biomedcentral.com/1472-6882/14/133/prepub
